# Cardiovascular magnetic resonance myocardial feature tracking predicts severity of wall motion abnormalities following acute coronary syndromes

**DOI:** 10.1186/1532-429X-15-S1-P200

**Published:** 2013-01-30

**Authors:** Kalpa De Silva, Asela Bandara, Andreas Schuster, Pablo Lamata, Roy Jogiya, Shazia T Hussain, Kaleab N Asrress, Nic Smith, Michael Marber, Eike Nagel, Simon Redwood, Divaka Perera, Sven Plein

**Affiliations:** 1 Cardiovascular Division, St. Thomas' Hospital, King's College London, London, UK; 2 Imaging Sciences, St. Thomas' Hospital, King's College London, London, UK; 3 Department of Computer Science, University of Oxford, Oxford, UK; 4 Multidisciplinary Cardiovascular Research Centre, Leeds Institute of Genetics, Health and Therapeutics, Leeds, UK

## Background

Left ventricular wall motion assessment following acute myocardial infarction (AMI) allows prediction of functional recovery. Current methods are based upon visual assessment, with inherent operator variability. CMR myocardial feature tracking (CMR-FT) is a recently introduced technique for tissue voxel motion tracking on standard steady-state free precession (SSFP) images to derive circumferential and radial myocardial mechanics. We sought to determine whether CMR-FT could be used as a quantitative measure of wall motion assessment and predict recovery in function following AMI.

## Methods

Patients presenting with non-ST elevation myocardial infarction (NSTEMI) were studied using a 3 Tesla Phillips Achieva system with Multi-transmit® technology and a 32-channel receiver coil, immediately prior to, and 3-months following, percutaneous coronary revascularization (PCI). Cine images were acquired using a SSFP cine technique, with analysis performed on the left-ventricular (LV) short-axis stack, covering the LV from apex to base. The acquisition pulse sequence provided a typical spatial resolution of 1.8 x 1.8 x 8 mm with a 2mm inter-slice gap and a temporal resolution of 50 frames per second. A 5-point wall motion scoring system was used to determine regional wall motion abnormalities (RWMA), with derivation of an indexed wall motion score (WMSI) specific to the region of infarction, according to the AHA 16-segment model. Myocardial strain parameters were derived following automated endo- and epi-cardial wall motion tracking of the SSFP cine short axis stack using dedicated software (Tomtec™, Germany). Pre-PCI LV short axis circumferential (Ecc) and radial (Err) peak strains were related to WMSI using a Pearson correlation analysis. Visual and quantitative analyses were performed by two blinded reviewers.

## Results

18 patients (Mean age 57±9 years, 78% male) with a mean Troponin T and LV ejection fraction (LVEF) of 1.6 ±1.1ug/L and 56±8% respectively completed the scanning protocol. Pre-PCI CMR-FT strain parameters were derived in all cases. Mean index WMSI was 2.57±0.03 reducing to 1.98±0.03 at follow-up (p=0.004). Mean index Ecc and Err were -23.1±15.5 and 21.0±15.5 respectively. Neither Ecc nor Err correlated with global LVEF (R=0.10, p=0.69 and R=-0.03, p=0.89, respectively). However, Ecc (R=0.51, p=0.04) and Err (R=-0.50, p=0.04) showed a linear and inverse linear correlation, respectively, with the regional WMSI. In addition, index Ecc correlated with the 3-month WMSI (R=0.55, p=0.03).

## Conclusions

CMR-FT may be used to quantify severity of wall motion following an ACS. Circumferential strain parameters are more accurate in predicting infarct related wall motion abnormalities and recovery in function.

## Funding

Heart Research UK

